# Development of Test Cases for Automated Vehicle Driving Safety Assessment Using Driving Trajectories

**DOI:** 10.3390/s24247981

**Published:** 2024-12-13

**Authors:** Woori Ko, Minkyu Shim, Sangmin Park, Soomok Lee, Ilsoo Yun

**Affiliations:** 1 Department of Transportation Engineering, Ajou University, Suwon 16499, Republic of Korea; go6017v@ajou.ac.kr; 2 Mobility Department, TÜV SÜD Korea Ltd., Seoul 07326, Republic of Korea; min-kyu.shim@tuvsud.com; 3 Department of Road Transport Research, The Korea Transport Institute, Sejong 30147, Republic of Korea; 4 Department of AI Mobility Engineering, Ajou University, Suwon 16499, Republic of Korea; soomoklee@ajou.ac.kr; 5 Department of Transportation System Engineering, Ajou University, Suwon 16499, Republic of Korea; ilsooyun@ajou.ac.kr

**Keywords:** test cases, driving safety, assessment, trajectory, automated vehicles

## Abstract

For consumers to have confidence in the safety of automated vehicles (AVs), AVs must be assessed using systematically developed scenarios to verify driving safety and reliability. In particular, verification using scenarios has been widely performed for the assessment and certification of AVs. This study aims to develop test cases based on driving trajectories to assess the driving safety of AVs. To achieve this, concrete scenarios were systematically developed from functional and logical scenarios. Drone video data analysis was conducted to extract representative lane-change trajectories for AVs on expressway ramp sections. Subsequently, the test cases were selected from concrete scenarios through simulations using time-to-steer (TTS). Finally, the effectiveness of utilizing trajectories for scenario-based driving safety assessments was verified. Furthermore, it is expected that this approach can be applied to other driving patterns by providing a detailed procedure for the test case developed in this study.

## 1. Introduction

The driving of automated vehicles (AVs) directly affects the safety of all objects and people in the surrounding environment, such as other vehicles, pedestrians, and bicycles, as well as the occupants inside the vehicle. Therefore, it is important to ensure the driving safety of AVs and to establish their reliability on roads. Assessment and certification phases verify the driving safety and reliability of AVs [1]. A scenario-based approach is commonly applied in the assessment and certification phases using the scenario framework proposed by the German PEGASUS project. The PEGASUS project divides scenarios into four levels according to their degree of abstraction—functional, logical, concrete, and test cases—and gradually refines them [2]. Each scenario represents a situation for assessment, provides descriptions of the range of parameters for assessment, sets the parameter values to be used for the assessment, and represents a scenario that yields meaningful results for safety assessment [3]. Among the four levels, the test case is the most practical scenario that can be directly utilized to assess the driving safety of AVs. In test cases, all experimental conditions, such as situations, each object’s behaviors, and the surrounding conditions, are clearly defined [3]. Hence, the development of test cases plays an important role in scenario-based assessments, and developed test cases are utilized in simulations and proving ground (PG) assessments [4].

To develop a more systematic and effective scenario for assessing the driving safety of AVs, it is essential to ensure compliance with the following three aspects: First, AVs must be able to adapt to various conditions and respond appropriately to environmental changes. Second, it is important to enable AVs to interact seamlessly with other nearby vehicles. This requires the ability to accurately interpret and predict the behaviors and intentions of other vehicles and make informed decisions. Finally, it is essential to thoroughly assess and rectify any technological limitations and drawbacks that may arise during the development and deployment of AVs [5].

Assessing the safety of a vehicle under various conditions involves evaluating its ability to drive safely in all scenarios by considering factors such as road type, weather conditions, lighting, and driving characteristics. The assessment also involves assessing whether the AV is taking appropriate actions in the face of abrupt environmental changes such as lane changes, sudden stops of a preceding vehicle, or the appearance of obstacles. It is important for AVs to adapt to all driving conditions when interacting with other vehicles nearby.

In addition, it is imperative to recognize and address the technological constraints and deficiencies of AVs during the pre-commercialization evaluation phase. The scenario-based safety assessment demonstrates remarkable effectiveness as it facilitates the generation of diverse assessment conditions through the process of crafting and developing scenarios that encompass various situations. Furthermore, the utilization of scenarios also enables verification of whether AVs possess appropriate measures to effectively address the three aforementioned assessment aspects.

This study aimed to develop test cases to assess the driving safety of AVs based on vehicle trajectories. First, a group of concrete scenarios was developed from functional and logical scenarios through a systemic process. Subsequently, the driving performance of AVs was analyzed through MATLAB/Simulink simulation using the concrete scenarios. In the simulation, the lane-change behavior of AVs was presented using actual vehicle trajectories during the lane change. To generate vehicle trajectories, this study analyzed video data taken using drones. The test cases were selected from a group of concrete scenarios using an assessment index. Time-to-steer (TTS) was used as an assessment index to select the test cases. TTS can determine the risk of collision based on combinations of various experimental parameters representing specific driving situations. If the predetermined criteria were met, the concrete scenario was selected as the test case.

This study aims to establish test cases for assessing driving safety in scenarios in which an ego vehicle (i.e., automated vehicles) cuts in front of a target vehicle in an adjacent lane in an expressway ramp area. Therefore, the spatial scope was set as an expressway ramp section where lane-change maneuvers frequently occur owing to lane merging and splitting. Real drone video data were analyzed to generate lane-change trajectories, and relevant experimental parameters were derived to assess the safety of the scenarios.

## 2. Literature Review

In this study, research related to scenario-based assessment and vehicle trajectory generation methods was reviewed. First, research on the safety assessment of AVs and the definition of scenario systems was examined. Second, a comprehensive review for the purpose of generating trajectories, including the analysis of drone video data, as well as other approaches, was conducted.

### 2.1. Scenario-Based Assessment Related Research

Huang et al. [6] introduced various methods for assessing the functionality of AVs. The assessment methods include software, simulation, X-in-the-loop simulation (XILS), and real road tests. Therefore, researchers have argued that it is necessary to evaluate and validate AV functionality in various environments.

Szalay et al. [7] proposed using scenario-in-the-loop (SciL) for the evaluation of AVs. SciL is a digital twin technique that simulates realistic traffic scenarios using a simulator. SciL can be used for vehicle development, assessment, and verification. The researchers developed diverse scenarios representing realistic traffic situations using SciL concept. Furthermore, the researchers demonstrated the effectiveness of SciL by evaluating AVs using the scenarios.

Riedmaier et al. [8] introduced a scenario-based methodology to ensure the safety of automated driving systems (ADS). This approach involves a comparative evaluation of AVs in individual traffic situations through a virtual simulation. To achieve this, scenarios were constructed using real-world traffic data. The researchers assessed scenarios in the simulations. The final selection of scenarios was guided by the outcomes of simulation-based assessments.

De Gelder et al. [9] devised an assessment methodology for AVs that leveraged actual road traffic scenarios obtained from driving data. The researchers generated parameterized scenarios using probability density functions estimated from driving data. This approach enabled the realistic quantification of scenarios by reviewing deceleration and cut-in scenarios in the case studies.

Ko et al. [3] proposed a scenario generation framework to assess the driving safety of AVs. The researchers focused on establishing a comprehensive process for scenario development from a functional scenario to test cases by utilizing the four-phase scenario system (i.e., functional, logical, concrete, and test cases) of the German PEGASUS project. Their approach involved constructing detailed functional scenarios based on traffic accident reports and creating logical scenarios based on combinations of all ranges, as well as introducing a framework for selecting test cases through simulations as the final step.

Wishart et al. [10] proposed assessment metrics that quantitatively describe the driving safety performance of AVs. Various metrics have been proposed, including time-to-collision (TTC), post-encroachment time (PET), and collision incident (CI). In addition, the metrics measure the degree of risk of situations using on- and off-board data sources.

Li et al. [11] introduced AV-FUZZER, a fuzz testing framework, to systematically identify safety violations in AV systems. AV-FUZZER randomly generates system inputs and repeatedly executes AV safety violation scenarios. For effective testing during the fuzzing process, driving simulations and unique navigation algorithms are combined to identify potential risk situations. This approach complements scenario-based methods by enabling the detection of system-level vulnerabilities.

Tang et al. [12] analyzed the moderating effect of policy measures on the intention to adopt AVs in China. After evaluating the impact of policies based on survey data, the researchers found that policies play an important mediating role in the intention to adopt AVs and that specific policies are effective in increasing consumer trust and acceptance. This emphasizes the importance of policy design to promote the introduction of AVs.

### 2.2. Vehicle Trajectory Generation Related Research

To assess the driving safety of AVs in a lane-changing situation, it is necessary to define methods for generating the trajectories of AVs and surrounding vehicles. In this study, lane-change trajectories were analyzed using drone video data. Therefore, related research on defining vehicle trajectories and analyzing drone video data was reviewed to derive insights.

Patel et al. [13] developed lane-change trajectories for high-dimensional automated driving functions using high-order polynomials to generate vehicle movement trajectories. Comparing various high-degree equations for the lane-changing trajectory of ego vehicles, it was found that sixth-degree polynomials for lateral motion and fifth-degree polynomials for longitudinal motion yielded the best results. The generated trajectories were modeled and analyzed using MATLAB/Simulink simulations to improve safety and stability.

Karunakaran et al. [14] proposed a method for extracting vehicle movement trajectories in lane-change scenarios for AVs based on point-cloud data collected by LiDAR. The generated scenarios were represented in the OpenX format to facilitate easy reuse in System Under Test (SUT) assessments for verification. The simulated trajectories were compared with actual driving trajectories to demonstrate their effectiveness.

Liu et al. [15] collected real-world driving data from 60 drivers to assess the safety impact of the Responsibility-Sensitive Safety (RSS) model during lane changes. Driving data were obtained using GPS, cameras, and radar sensors from AVs. The researchers automatically identified cut-in situations that satisfied predefined longitudinal threshold conditions and extracted data such as position, speed, and acceleration to generate lane-change trajectories. The RSS-embedded Adaptive Cruise Control (ACC) model based on the extracted trajectories showed improved safety performance compared to human drivers and ACC stand-alone models.

Krajewski et al. [16] generated vehicle trajectories using a drone video dataset called HighD that focused on the mainline sections of German highways. Vehicle trajectories were created by post-processing the x- and y-positions, speeds, and accelerations extracted by connecting detections in consecutive frames. The generated individual vehicle trajectories were categorized into four types: free driving (no preceding vehicle influence), car following (with preceding vehicle influence), risky driving with a leading vehicle based on TTC and time headway (THW), and lane changes.

Moers et al. [17] generated vehicle trajectories using a drone video dataset called exiD, which focused on the entrance and exit sections of German autobahns. The researchers followed the methodology of Krajewski et al. [16] to generate directional bounding boxes for detected objects from video data. Subsequently, post-processing was performed to extract information such as position, direction, speed, acceleration, and lane information for each time stamp. The extracted vehicle trajectory information was used to create challenging scenarios.

### 2.3. Summary

By conducting an extensive literature review on scenario-based assessment, it was found that the scenario framework developed in the PEGASUS project has garnered significant recognition and adoption. The scenario framework established in the PEGASUS project employs a progressive approach that reduces the abstraction levels of the scenarios. According to Huang et al. [6] and Szalay et al. [7], the developed scenarios can be applied to various assessment environments, including simulations and virtual tests. Building upon these studies, this study focuses on developing scenarios for specific spatial conditions—lane changes on expressway ramp sections—emphasizing practical applicability. Riedmaier et al. [8], de Gelder et al. [9], and Ko et al. [3] developed methods for parameterizing scenarios based on driving data, generating the representative scenarios as test cases. The present study leverages their approach while adding drone video data analysis to extract lane-change trajectories.

Additionally, Wishart et al. [10] emphasize the importance of quantitative safety performance metrics, such as TTC, PET, and CI. This manuscript adopts TTS as its primary assessment index, leveraging its suitability for assessing lane-change maneuvers in expressway test cases. The application of TTS represents a certain level of safety in selected test cases.

In previous studies, the definition of vehicle driving trajectories and analysis of drone data have been extensively explored. Patel et al. [13] and Liu et al. [15] utilized high-degree polynomials and driving data, respectively, to model and validate lane-change trajectories. In contrast, this study analyzes drone video data to represent realistic and statistically derived trajectories for AV assessments. This study distinguishes itself by emphasizing the development of a lane-change assessment test case for driving safety verification, rather than the development of AVs aimed at commercialization. Karunakaran et al. [11] analyzed LiDAR data to derive lane-change trajectories for general vehicles. This deviates from Karunakaran et al. [11] in terms of the foundational data source for trajectory generation, employing an analysis of drone video data to extract trajectories, thus presenting a unique approach to the subject matter.

Furthermore, Krajewski et al. [16] and Moers et al. [17] used drone datasets to explore general highway scenarios involving individual vehicle driving trajectories. However, in this study, the statistically analyzed lane-change trajectories of individual vehicles were combined to provide the most representative trajectory as a scenario, providing a unique approach. The use of a representative trajectory in this study was motivated by the expectation that AVs should prioritize safety and avoid abrupt and drastic lane changes. By generating and analyzing these trajectories, specific test cases were selected based on an assessment index. Notably, this study focused on lane-change situations on the expressway ramp section and was conducted through simulations, adding another distinct aspect to the research.

In summary, this study synthesizes the findings of prior studies and builds upon them to establish a robust and replicable methodology for AV safety assessment. The use of drone video data and the targeted focus on expressway ramp sections represents a critical step toward bridging the gap between theoretical scenario generation and practical AV safety assessment. By selecting representative trajectories and developing them into test cases, this study contributes to both the methodological and practical advancements of AV safety research.

## 3. Trajectory-Based Test Case Development Methodology

### 3.1. Drone Video Data Analysis Method

Drone video data of the exit ramp sections on expressways were used to extract lane-change trajectories. Multiple exit ramp sections were recorded using a drone to obtain sufficient lane-change trajectory data from the expressway mainline to the exit lane. Using the drone video data, vehicles that underwent lane changes were first identified through visual inspection. Subsequently, key parameters such as the time and longitudinal distance required for individual vehicles to complete lane changes were calculated and statistically analyzed to identify representative lane-change patterns.

Specifically, drone video data were recorded between November and December 2016. The recording durations for each section were as follows: 60 min at the Nooji Junction (JC) of the Sudogwon Je1sunhwan Expressway in the direction of Ilsan, 20 min in the morning and 25 min in the afternoon at the Osan Interchange (IC) of the Gyeongbu Expressway, and 25 min at the Gamgok IC of the Jungbunaeryuk Expressway. The maximum road speed limit for each section is 100, 110, and 110 kph, respectively. The recorded traffic volumes in each section were 4335 vehicles at Nooji JC, 3303 vehicles at Osan IC, and 998 vehicles at Gamgok IC. Among these, lane-change analyses were conducted for 108 cases at Nooji JC, 90 cases at Osan IC, and 17 cases at Gamgok IC.

**Figure 1:**
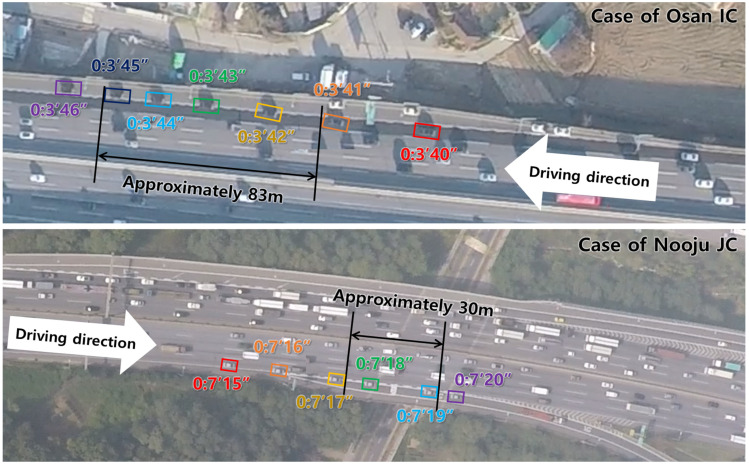
Snapshot examples of lane-change trajectory.

The Korean expressway system designates lane markings with a painted length of 10 m and a gap length of 10 m [18]. Therefore, this rule was used to estimate the longitudinal distance required for lane change. When visually inspecting the drone video data, the lane-change trajectories can be represented as shown in Figure 1.

After performing the analysis for all lane-change cases, as shown in Figure 1, the lane-change duration time and the length of individual vehicles were calculated. Through statistical analysis, the mean, median, and standard deviation for the lane-change duration and length of vehicle were calculated. Finally, the median values were selected to generate a representative lane-change trajectory. Note that lane-change trajectories from human-driven vehicles were utilized in this study. This is because of the resource limitations in collecting real AV driving data. Although the lane-change trajectory used in this study may not fully represent the driving trajectory of AVs, it can be used to estimate the lane-change trajectories for general vehicles. In the long term, realistic AV trajectories can be achieved by adjusting the trajectories based on actual AV driving data.

### 3.2. Driving Trajectory Generation Method

**Figure 2:**
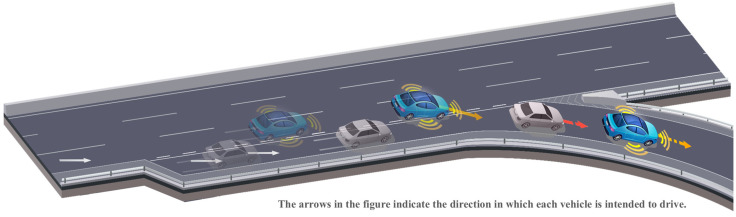
Overall flow of cut-in scenario on expressway ramp section.

Figure 2 shows the scenarios implemented in this study. Specifically, a group of test cases was developed in which an ego vehicle (AV) cuts in front of a target vehicle in an adjacent lane in an expressway ramp area. The primary focus of this safety assessment was to verify the performance of the AV when the target vehicle was strategically deployed at diverse speeds and locations. In Figure 2, the blue-colored vehicle represents the ego vehicle, and the gray-colored vehicle is the target vehicle, with the transparency of the vehicles changing over time to represent their approximate positions. An assessment was performed to determine whether the ego vehicle could change lanes without causing collisions. To achieve this, a general lane-change trajectory selected from the analyzed drone video data was created using the automated driving toolbox in MATLAB/Simulink. The lane-change model used a cubic spline model, the default model built into MATLAB/Simulink. If collisions are inevitable, further studies will be needed to assess whether the system can respond to minimal risk conditions (MRC) by aborting the lane change.

**Figure 3:**
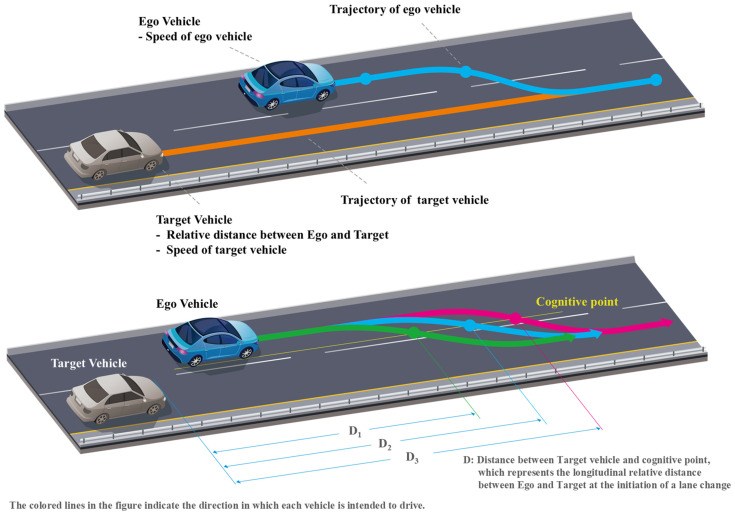
Example of cut-in driving trajectory generation in Matlab/Simulink.

The lane-change trajectory configuration in MATLAB/Simulink enables the individual definition of driving points for each object, facilitating the placement of diverse points on a path, as depicted in Figure 3. Following the placement of these points, the objects traverse the trajectory determined by the designated points. In Figure 3, the cognitive point represents the longitudinal relative distance between two vehicles at the initiation of a lane change. By placing the cognitive points at various positions such as D_1_, D_2_, and D_3_, the different longitudinal relative distances between the two vehicles can be assessed. Subsequently, the ultimate lane-change trajectory for the ego vehicle can be determined by leveraging the analysis results from the drone video data.

### 3.3. Logical Scenario Generation for Ramp Section Lane Change

The scenario development process followed the method described in Ko et al. [3]. The logical scenario establishes the types and ranges of parameter values related to a specific situation presented in the functional scenarios [3]. Figure 2 presents a visual representation of the functional scenario. Therefore, the types and ranges of the parameter values vary according to the functional scenario in Figure 2. The parameters related to the cut-in situation of the ego vehicle on the expressway ramp section are listed in Table 1. The parameters in Table 1 were selected based on the results of a survey conducted with professionals engaged in the development and assessment of AVs while considering the feasibility of model implementation. Consequently, the trajectories and longitudinal velocities of the ego and target vehicles, as well as the relative distance between the two vehicles, were considered to delineate the behavior of the ego and target vehicles.

The parameter ranges are listed in Table 1. The maximum value of the longitudinal velocity of the ego vehicle was established from the speed limit on the expressway ramp sections, with the assumption of adherence to regulatory requirements. The longitudinal velocity of the ego vehicle was incremented in steps of 10 km/h for experimental purposes. The range of longitudinal velocities for the target vehicle was established by setting the maximum value to the 99th percentile of the traffic speed data obtained from the vehicle detection system (VDS) implemented on the expressway. The relative distance between the two vehicles was set according to the minimum distance specified in UN Regulation No. 157. UN Regulation No. 157 is an international regulation that addresses the approval and assessment of vehicles equipped with automatic lane-keeping systems; it also provides guidelines for conducting assessments, including various items, such as the minimum following distance, driving speed, deceleration, and lateral acceleration [19]. Particularly, the minimum following distance increases at 0.1 s intervals from 1.0 s to 1.8 s.

### 3.4. Safety Assessment Indicators and Criteria Setting

**Figure 4:**
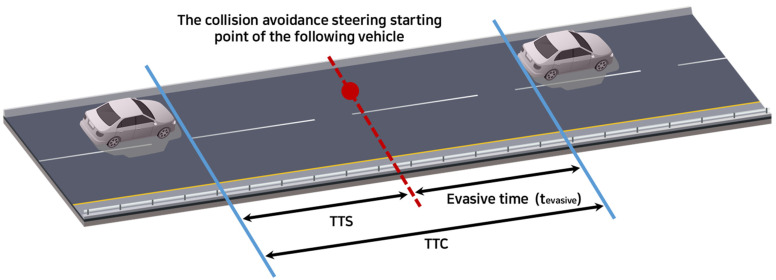
The concept of TTS based on TTC.

Test cases were designed to explore various combinations of variables that led to significant outcomes in terms of an AV’s ability to avoid collisions under specific assessment constraints. The term “significant outcome” refers to the ability of the AV to satisfy the assessment criteria for driving safety in a particular scenario. Specific assessment indices and criteria for assessing driving safety exist for a variety of situations, and only scenarios that meet these criteria are classified as test cases. Widely used safety assessment indices include time-to-collision (TTC), time-to-maneuver (TTM), time-to-steer (TTS), time headway, and conflict index (CI). Because this study models cut-in situations, TTS was selected as a suitable safety assessment index. TTS represents the time required for steering to avoid a collision with a preceding vehicle [20] and can be mathematically defined as shown in Figure 4 and Equation (1) [21]:

(1) $$TTS=TTC-t_{eva}$$

$$t_{eva}\left(y_{eva};a_{eva}\right)=\sqrt{\frac{2y_{eva}}{a_{eva}}}+\tau_{s}$$

where

TTS: time to steer
TTC: time to collision
$t_{eva}$: evasive time
$y_{eva}$: lateral position (determined by the distance between the two vehicles’ centers)
$a_{eva}$: lateral acceleration
$\tau_{s}$: steering loss time.

To calculate TTS, $t_{eva}$ must first be determined. Ackermann et al. [21] assumed $\tau_{s}$ to be 0.1 s. Similarly, in this study, $\tau_{s}$ was assumed to be 0.1 s. $y_{eva}$ corresponds to the distance between the two vehicles’ centers. Assuming that two vehicles travel along the center of each lane, $y_{eva}$ becomes the lane width. Because the spatial range of this study was an expressway ramp section, $y_{eva}$ was set to 3.6 m in accordance with the standard expressway lane width in Korea. In addition, for $a_{eva}$, the maximum lateral acceleration standard defined in UN Regulation No.79 was used [22]. According to the literature, the maximum lateral acceleration value suggested for passenger cars and small trucks is 3 m/s^2^ and for other heavy vehicles, is 2.5 m/s^2^ [22]. Therefore, in this study, 3 m/s^2^ was set as the $a_{eva}$ value. Using the above values, the final TTS is expressed by Equation (2).

(2) $$t_{eva}\left(y_{eva};a_{eva}\right)=\sqrt{\frac{2y_{eva}}{a_{eva}}}+\tau_{s}=\sqrt{\frac{2\times 3.6}{3}}+0.1\approx 1.649$$

$$TTS=TTC-t_{eva}=TTC-1.649$$

The reason TTS was selected as the safety assessment index is to account for the duration required by both the ego and target vehicles to complete a lane-change maneuver smoothly without encountering any collisions. The specific TTS threshold of over 0.4 s and under 1.0 s was established by referencing previous research findings [19,23]. If the TTS falls below 0.4 s, it is deemed necessary for the ego vehicle to relinquish the lane-change attempt and transition to the minimum risk condition (MRC). Conversely, if the TTS exceeds 1.0 s, the scenario is regarded as lacking significant risk and is subsequently excluded from the selection of test cases.

## 4. Trajectory-Based Test Case Development Results

### 4.1. Drone Video Data Analysis Results

Drone video data of expressway exit ramp sections were utilized to extract trajectories. Only the trajectories of lane changes from the mainlines to the deceleration lanes were extracted using visual inspection, resulting in the observation of 215 lane changes from three data collection locations: Nooji JC, Osan IC, and Gamgok IC. The lane-change duration time ranged from a minimum of 2 s to a maximum of 6 s, with data exceeding 4 s considered as outliers and excluded from the analysis. Lane-change distances ranged from a minimum of 25 m to a maximum of 120 m. Data exceeding 80 m were considered outliers and removed from the analysis. Table 2 displays the representative values of lane-change time (s) and lane-change distance (m) for 168 lane-change trajectories after the exclusion of outlier data points. Finally, the median value of distance required to change lanes was selected to generate a representative lane-change trajectory.

### 4.2. Implementation of Concrete Scenarios

A concrete scenario was generated by combining various experimental values within the range set for each parameter type within the logical scenario in Table 1 according to the vehicle trajectory generated based on the situation. After the trajectory and experimental values were set, the risk of collision was classified by calculating the TTS, which is the criteria for selecting the test cases. TTS served as a safety assessment tool for selecting appropriate test cases among the concrete scenarios generated in the experiment. Because risk is a judgment based on the overall situation, TTS is calculated from the perspective of the target vehicle and not the ego vehicle. Therefore, the time required for the following target vehicle to steer to avoid a collision in response to the ego vehicle’s cut-in maneuver was calculated.

### 4.3. Test Case Selection Results

In total, 648 concrete scenarios were generated by combining the parameter ranges specified for each parameter type in Table 1. These concrete scenarios were obtained by multiplying all possible values within the assessment ranges of the parameters. This encompassed six speed values for the ego vehicle, twelve speed values for the target vehicle, and nine values representing the relative distance between the two vehicles. However, note that when the target vehicle speed is relatively low, the ego vehicle can safely execute lane changes without the risk of collision. Therefore, only scenarios in which the target vehicle’s speed exceeded that of the ego vehicle were considered valid for assessment. Consequently, 39 combinations with a target vehicle speed higher than that of the ego vehicle, along with all 9 combinations of relative distances, were combined, resulting in 351 scenarios for modeling.

**Figure 5:**
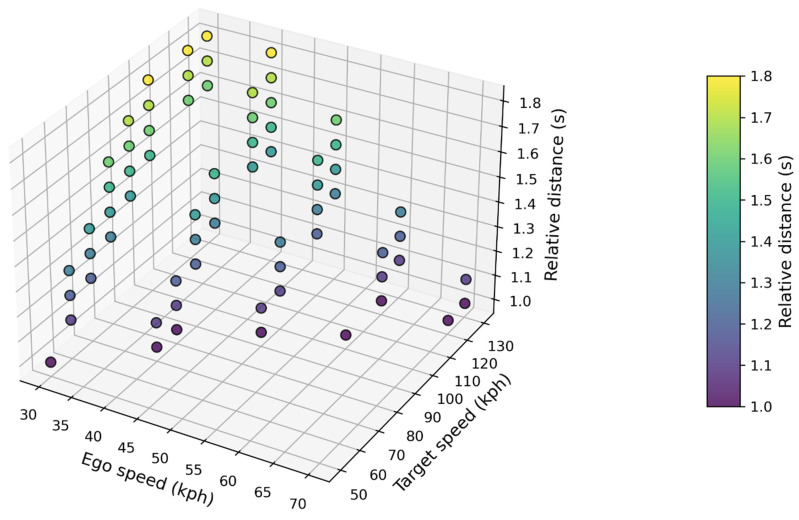
Scatter plot of selected 68 test cases.

Subsequently, the simulation results were rigorously analyzed by employing predefined safety assessment criteria derived as an outcome of the methodology development process. Only scenarios that met the condition of TTS being greater than 0.4 s and less than 1.0 s were considered. Based on the criteria, 68 test cases were selected; the simulation results are presented in Figure 5 and Table 3. In particular, Figure 5 depicts a scatter plot of scenarios with TTS values ranging from 0.4 to 1.0 s. The x-axis represents the speed of the ego vehicle, the y-axis represents the speed of the target vehicle, and the z-axis and colored dots represent the relative distance between the two vehicles.

To illustrate an example of the selected test cases, let us consider the 20th test case in Table 3. In this scenario, the ego vehicle travels at a speed of 40 km/h, while the target vehicle travels at a speed of 90 km/h, with a relative distance of 1.2 s between the two vehicles. TTS values between 0.4 and 1.0 s allow this scenario to be selected as the test case. This particular scenario was chosen as the test case because the target vehicle approaches the ego vehicle at a higher speed, and the relatively short distance between the two vehicles increases the risk of collision during the lane-change maneuver of the ego vehicle.

Based on the selected test case in Table 3, the trend of the relative distance and speed difference between the two vehicles can be observed. Even at the same relative distance, the relative speed is low when the speed of the two vehicles is low, but the relative speed increases when the speed of the two vehicles is high. In addition, a scenario with a large speed difference between the two vehicles can also be used in the assessment if the relative distance increases. In the selected test case, it is possible to efficiently evaluate driving safety based on whether the vehicle properly performs the lane change and how it responds in an accident-risk situation.

## 5. Conclusions

The aim of this study was to ensure the driving safety of AVs and to build confidence in their performance on the road. To achieve this, a scenario-based assessment was employed, aimed at developing test cases. As an example of test case development, the spatial scope was limited to expressway exit ramp sections for lane-change situations. Subsequently, statistical analysis was conducted to analyze the duration time and distance required for lane change using drone video data. Based on the median value of distance required for lane changes, vehicle trajectories were generated and utilized in a simulation modeling AVs. To simulate the scenarios, a range of experimental parameters related to cut-in situations was selected. Experimental parameters within the selected range were combined to execute the simulations. The criteria for safety assessment used in the selection of test cases was TTS between 0.4 and 1.0 s, based on a literature review. As a result of the validation process, an initial set of 648 scenarios was refined, resulting in 68 test cases being finally selected. This meticulous curation enhances assessment efficiency by extracting pertinent assessment scenarios from a vast number of potential scenario combinations. The simulation outcomes of the test cases conducted in this study demonstrated the efficacy of employing real vehicle trajectories for safety assessments. The proposed methodology not only strengthens safety evaluations as part of the AV safety certification process but also enables the integration of experimental values into real-world scenarios for lane-change safety assessments. Moreover, this approach can be used as a basis for establishing safety certification guidelines specific to automated driving safety in lane-change situations, highlighting its practical applicability. The selected test cases represent scenarios in which there may be a risk of collision when the AV performs a cut-in under certain conditions and can be utilized for a cut-in performance assessment to assess the vehicle’s ability to respond safely under such conditions. Additionally, based on the methodology of this study, test cases can be developed for different scenarios if appropriate safety assessment indicators are established for different situations.

The limitation of this study is that the trajectory estimation of AVs during lane-change scenarios is based on the behavior of non-automated vehicles. The driving behavior of human-driven vehicles and automated vehicles will be different, and as a result, the test case output may also be different. However, currently, the acquisition of AV trajectory data is difficult because AVs are not sufficiently commercialized and are undergoing development. Therefore, future study needs to further improve accuracy by leveraging trajectory data from AVs to improve the model. Another limitation is that only two vehicles are considered. In this study, simulation focused solely on the interaction between the preceding ego vehicle and the following target vehicle to verify the AV safety performance in the most basic situation. However, on real-world roads, the situation becomes more complicated due to the various vehicles that exist around AVs, so further research needs to be conducted reflecting this. Furthermore, there is a limitation in that this study has only focused on lane changes in the expressway ramp section. As the methodology is still in the developmental stage, the model was verified through simulation by focusing on one situation. In the future, it will be necessary to conduct test case development research that considers various situations to complement the results of this study.

## Figures and Tables

**Table 1:** Definition of parameters and ranges.

Parameters		Ranges		
		Minimum Value	Incremental Value	Maximum Value
Ego Vehicle	Speed (km/h)	30	10	80
	Driving trajectory	Cuts-in to ramp exit lane with a single trajectory		
Relative distance between Ego and Target (s)		1.0	0.1	1.8
Target Vehicle	Speed (km/h)	10	10	130
	Driving trajectory	Going straight ahead on ramp exit lane with a single trajectory		

**Table 2:** Drone data analysis result excluding outliers.

Type	Ranges				
	Data Count	Mean	Median	Mode	Standard Deviation
Time required to change lane (s)	168	2.36	2.00	2.00	0.58
Distance required to change lane (m)	168	43.91	41.70	30.00	12.40

**Table 3:** Parameters of selected 68 test cases.

Test Case No.	Ego Speed (kph)	Target Speed (kph)	Relative Distance (s)	Test Case No.	Ego Speed (kph)	Target Speed (kph)	Relative Distance (s)
1	30	50	1.0	35	30	80	1.4
2	40	70	1.0	36	30	90	1.4
3	40	80	1.0	37	40	90	1.4
4	50	90	1.0	38	40	100	1.4
5	60	100	1.0	39	40	120	1.4
6	60	120	1.0	40	40	130	1.4
7	70	120	1.0	41	50	120	1.4
8	70	130	1.0	42	50	130	1.4
9	30	60	1.1	43	30	80	1.5
10	40	70	1.1	44	30	90	1.5
11	40	80	1.1	45	30	100	1.5
12	50	90	1.1	46	40	100	1.5
13	50	100	1.1	47	40	120	1.5
14	60	120	1.1	48	40	130	1.5
15	60	130	1.1	49	50	120	1.5
16	70	130	1.1	50	50	130	1.5
17	30	60	1.2	51	30	80	1.6
18	30	70	1.2	52	30	90	1.6
19	40	80	1.2	53	30	100	1.6
20	40	90	1.2	54	30	120	1.6
21	50	100	1.2	55	30	130	1.6
22	50	120	1.2	56	40	120	1.6
23	60	120	1.2	57	40	130	1.6
24	60	130	1.2	58	50	130	1.6
25	30	60	1.3	59	30	90	1.7
26	30	70	1.3	60	30	100	1.7
27	30	80	1.3	61	30	120	1.7
28	40	90	1.3	62	30	130	1.7
29	40	100	1.3	63	40	120	1.7
30	50	100	1.3	64	40	130	1.7
31	50	120	1.3	65	30	100	1.8
32	50	130	1.3	66	30	120	1.8
33	60	130	1.3	67	30	130	1.8
34	30	70	1.4	68	40	130	1.8

## Data Availability

The data used in this study can be made available.
